# Goal-directed therapy in the perioperative management: is a complete hemodynamics bundle of care better?

**DOI:** 10.1186/s13054-021-03527-3

**Published:** 2021-03-16

**Authors:** Nicolas Herzog, Jean-Baptiste Dablin, Christophe Giacardi, Marc Danguy des Déserts

**Affiliations:** 1 Federation of Anesthesiology and Intensive Care Unit, Clermont-Tonnerre Military Training Hospital, Brest, France; 2 Department of Anaesthesiology and Surgical Intensive Care, Centre Hospitalier Et Universitaire de Brest - Université de Bretagne Occidentale, Brest, France

We read with a deep interest Messina et al.’s meta-analysis focusing on goal-directed therapy (GDT) in major visceral/non-cardiac surgery [1]. This research describes a reduction in perioperative complications in favor of GDT therapy, but not an improvement in perioperative mortality. Although the article supports the use of GDT in the perioperative setting, optimal GDT protocol still remains unclear.

The 21 studies included in the analysis can be divided into two subgroups: on the one hand those which only protocolized the fluid management and on the other hand those which protocolized a complete hemodynamics bundle of care including fluid management but also vasopressor or inotrope use. The second subgroup of studies (complete bundle of care) appears to have the most beneficial effect on perioperative complications found in the meta-analysis. This subgroup also included five of the only six studies of the meta-analysis which found a significant effect of GDT on perioperative complications by themselves. Moreover, the two studies with the greatest benefit of GDT also included a mean arterial pressure goal and the use of vasopressors and dobutamine in their protocols [2, 3].

In our opinion, the additional benefit of bundle protocols could be explained by the reduction in hypotension episodes occurrence and duration, thanks to a more frequent screening and a more aggressive treatment of these episodes in such protocols. In fact, even if the optimal blood pressure target remains controversial, hypotension is a well-known risk factor for complications in the perioperative period [4]. To corroborate this hypothesis, some GDT protocols which included a blood pressure target and interventions to reach it have shown a reduction in hypotension episodes or higher mean arterial pressure levels during the intraoperative period [2, 3].

In conclusion, all GDT protocols are not equivalent, and complete hemodynamics GDT protocols seem to be more efficient than fluid management only protocols. We suggest exploring the possible benefit of complete hemodynamics GDT protocols on morbidity and mortality in major visceral and non-cardiac surgery.

## Data Availability

Data sharing is not applicable to this article as no datasets were generated or analyzed during the current study.
